# Alginic Acid from *Padina boryana* Abate Particulate Matter-Induced Inflammatory Responses in Keratinocytes and Dermal Fibroblasts

**DOI:** 10.3390/molecules25235746

**Published:** 2020-12-05

**Authors:** Thilina U. Jayawardena, K. K. Asanka Sanjeewa, Lei Wang, Won-Suk Kim, Tae-Ki Lee, Yong-Tae Kim, You-Jin Jeon

**Affiliations:** 1 Department of Marine Life Sciences, Jeju National University, Jeju 690-756, Korea; tuduwaka@gmail.com (T.U.J.); asanka.sanjeewa001@gmail.com (K.K.A.S.); comeonleiwang@163.com (L.W.); 2 Marine Science Institute, Jeju National University, Jeju Self-Governing Province 63333, Korea; 3 Department of Pharmaceutical Engineering, Silla University, Busan 46958, Korea; wskim@silla.ac.kr; 4 Department of Hotel Cuisine & Baking, Jeonnam State University, Jeonnam 57337, Korea; tglee@dorip.ac.kr; 5 Department of Food Science and Biotechnology, Kunsan National University, Gunsan 54150, Korea

**Keywords:** *Padina boryana*, alginic acid, particulate matter, skin, inflammation, chelation

## Abstract

Particulate matter (PM) is a significant participant in air pollution and is hence an inducer of serious health issues. This study aimed to evaluate the dust protective effects of alginate from *Padina boryana* (PBA) via inflammatory-associated pathways to develop anti-fine dust skincare products. In between the external and internal environments, the skin is considered to be more than a physical barrier. It was observed that PM stimulates inflammation in the skin via activating NF-κB and MAPK pathways. The potential of PBA to inhibit the studied pathways were evident. The metal ion content of PM was considerably reduced by PBA and thus attributed to its chelation ability. Current research demonstrated the potential of *P. boryana* alginates to be implemented as a protective barrier against inflammation imposed with heavy metal and bacterial-derived endotoxin bound to the surface of the PM. Concisely, the results suggest that the bioactive components derived from the brown algae *Padina boryana* increased the cellular resistance to PM-stimulated inflammation-driven skin damage.

## 1. Introduction

Air pollution is supported via particulate matter (PM) and is comprised of a heterogeneous mixture of components. These include volatile particles, organic matter, metals, and ionic material [1]. The composition of the mixture varies depending on the source of generation. Both anthropogenic and natural sources contribute to this phenomenon. The PM could cause health issues due to its accumulation in the atmosphere. Pulmonary toxicity, as well as skin irritations, are possible considering their constituents. Several studies have been conducted regarding PMs’ effect on the respiratory system. Fernando et al. (2017) report on the influence of ERM-CZ100 (organic constituent fine dust) and ERM-CZ120 (inorganic constituents) on RAW macrophages and inflammation induction [2]. RAW macrophages were further assessed against CRM No.28, considering pulmonary issues and taking it as a model by Jayawardena et al. (2018) [3].

When considering the literature, two distinct sources of PM in the induction of inflammation are emphasized. Several reports account for the source to be the transition metal ion content which influences the inflammation via an oxidative stress pathway. The oxidative stress arises due to the Fenton chemistry pathway-implicated radicals and are made responsible by some authors [4,5]. The oxidative mechanism is most applicable to the smaller sized PM, in which it possesses a higher surface area and a large number of particles. These have been evident to be higher in toxicity compared to their larger counterparts [6]. The second suggestion is the bacteria-derived endotoxin bound to the surface of the particle causing the inflammation stimulation [7,8].

The skin is the outermost barrier of the body and is susceptible to alien factors and these could cause inflammatory disorders. For this reason, anti-inflammatory agents applicable to the skin are important. A distinct role of the skin is to provide immunity against foreign matter and to become a critical point between the external and internal environments. Even though it can individually act as an immunological organ, its effective function is observed with the resources supported by the immune system. A major component of the skin is its outermost keratinocyte layer. It performs immune function via the production of cytokines and by responding to cytokines. The dermal component of the skin contains fibroblasts. This is traditionally not considered a component of the immune system. But recent research suggests the crosstalk between the keratinocytes and the fibroblasts significantly contributes to maintaining the homeostasis of the skin immune system [9]. These generate secondary cytokines such as IL-6. It was reported during wound healing that the dermal fibroblasts contribute as a major source of keratinocyte growth factor (KGF) [10].

The present study was conducted focusing on the effect of PM (CRM no. 28) on the skin cells. Selectively, the keratinocytes and the inner layer fibroblasts extracted alginic acid from *P. boryana* to inhibit the inflammation-induced via PM. Marine algal polysaccharides have received much attention due to their high availability and ability in sustainable use as well as their biocompatibility. Alginate, which is a polymer comprised mainly of β-D-mannuronic acid and α-L-guluronic acid, forms hydrogels, chelate metals and performs anti-inflammatory, anti-oxidant properties [11,12]. In this study, researchers believe that the effect of transition metal ions and bacteria-derived endotoxin bound to the surface of the PM causes inflammation. Therefore, it aims to evaluate the potential of *P. boryana*-derived alginate to counteract the effect caused by PM. This could open up the sustainable usage of naturally derived phytochemicals as candidates for formulating skin care products against PM-induced skin damage.

## 2. Results

### 2.1. Proximate Composition and Chemical Composition

The proximate composition results provide a better understanding of the nutritional components of the selected seaweed. Accordingly, *P boryana* consists of a higher amount of crude polysaccharides (57.87 ± 0.63). Crude proteins are also present in the sample (16.36 ± 0.32). The ash content was reported as 14.14 ± 0.72, symbolizing higher mineral content due to its natural habitat. The moisture content was 6.2 ± 0.54, while the lipid content was reported as the lowest (1.03 ± 0.25). Table 1 indicates the chemical composition of the purified alginate (PBA).

### 2.2. Structural Characterization of PBA

**Figure 1:**
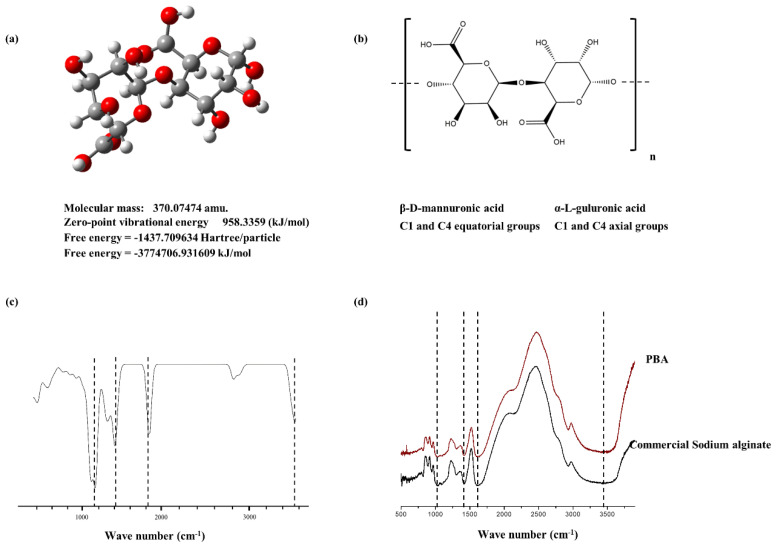
Characterization of alginic acid. (**a**) Structure of constructed dimeric unit of alginic acid 3D, (**b**) Skeletal formula of the alginic acid dimer with its monomeric units representing stereochemistry in 2D, (**c**) Vibrational spectra of alginic acid dimer calculated and constructed with density functional quantum chemical (DFT) calculations using B3LYP level, 6–31G (d,p) basis set, (**d**) FTIR spectroscopic analysis of PBA compared with commercial sodium alginate.

The structure of the PBA was characterized by FTIR analysis (Figure 1d). Distinct peak patterns were referred to with the commercial sodium alginate available. The prominent peaks were also referred to with the early reports published. It was confirmed that PBA well aligns with the commercial level alginic acid. O-H stretching vibrations were observed in the range of 3425 cm^−1^. Carboxylic group stretching vibrations were visible in 1680 cm^−1^ and 1420 cm^−1^ [13]. Furthermore, these data were referred to with the pre-defined spectral features obtained via computational quantum chemistry calculations. The constructed disaccharides were analyzed via Gaussian software to generate Cartesian coordinates. These were optimized using semi-empirical methods. This was subjected to the harmonic vibrational calculations with time-dependent density functional quantum chemical (DFT) theory using the B3LYP level, 6–31G (d,p) basis set. Figure 1a–c indicate the resulting structure geometry of dimers constructed, their free energies calculated and the vibrational spectroscopy obtained.

### 2.3. Potential of PBA to Reduce PM-Stimulated Inflammatory Responses in Keratinocytes and Fibroblasts

**Figure 2:**
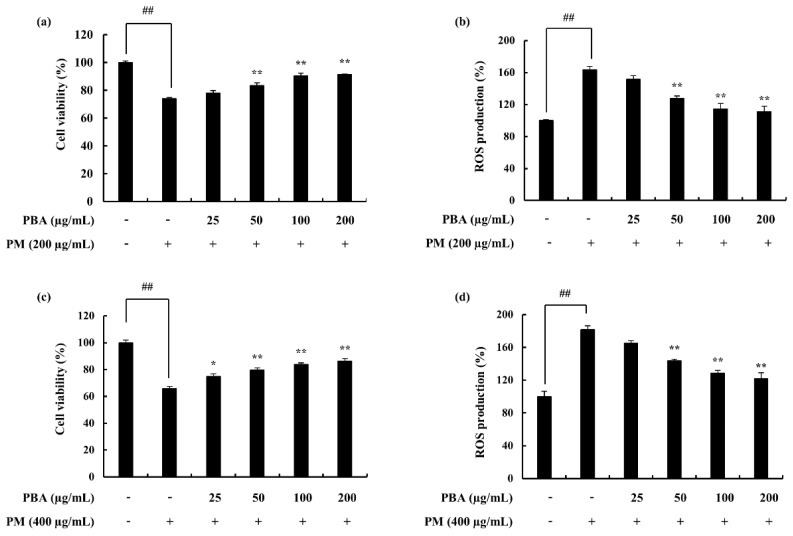
(**a**) PBA cytoprotective effect evaluation, (**b**) ROS production inhibition effect of PBA, in PM-induced keratinocytes, (**c**) Cytoprotective ability of PBA, (**d**) ROS production inhibition effect of PBA, in PM-induced HDF. Triplicated experiments were used to evaluate the data. Results are represented as mean ± SD; * *p* < 0.05, ** *p* < 0.01. (# denotes significance compared to control while * represents significance compared to the PM-treated group).

**Figure 3:**
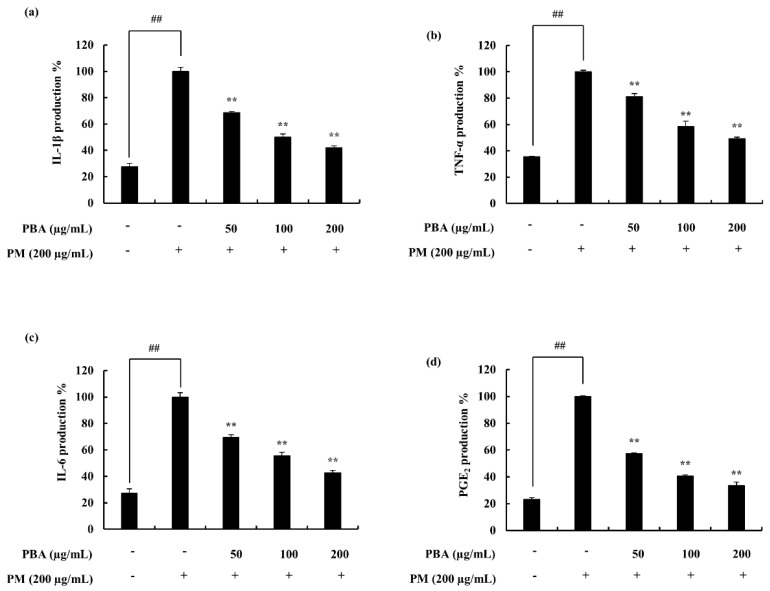
Effect of PBA on the keratinocytes and its production of inflammatory mediators (PGE2) including cytokines (IL-1β, IL-6, and TNF-α). Culture supernatants of RAW 264.7 cells after successful treatment of PM were used to quantify the inflammatory cytokines and PGE2. Triplicated experiments were used to evaluate the data and the mean value is expressed with ± SD. ** *p* < 0.01. (# denotes significance compared to control while * represents significance compared to PM treated group).

**Figure 4:**
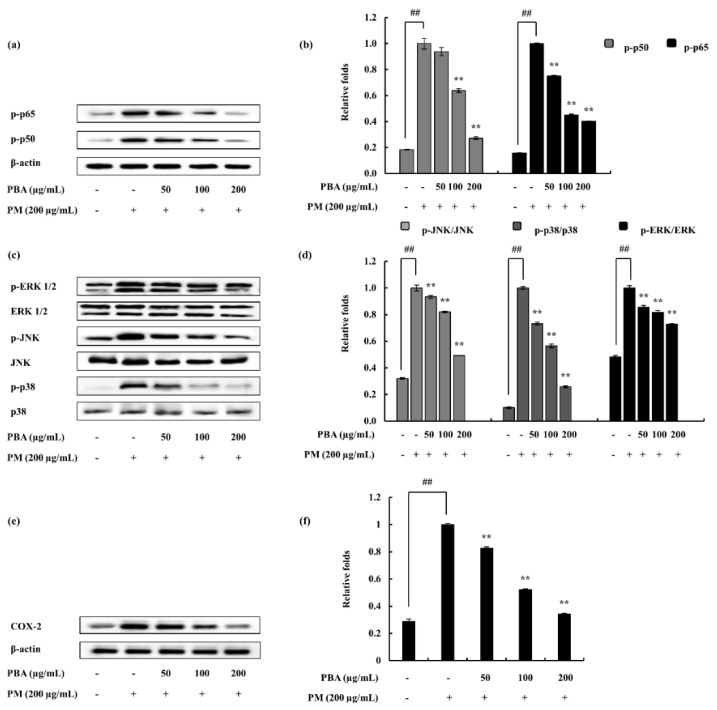
Effect of PBA on keratinocytes to inhibit NF-κB-associated signals, MAPK pathway molecules, and COX-2. (**a**) p50 and p65 in the cytosol, (**c**) p38, JNK and ERK, (**e**) COX-2 data determined using Western blotting. Quantitative data were analyzed using ImageJ software (**b**,**d**,**f**). Results are expressed as the mean ± SD of three separate experiments. ** *p* < 0.01. (# denotes significance compared to control while * represents significance compared to the PM-treated group).

The treatment of PBA resulted in the recovery of the cell viability, which was downregulated due to the stimulation of PM in keratinocytes and fibroblasts. ROS level in PM-stimulated keratinocytes was significantly declined in the 50, 100, 200 µg/mL concentrations where 25 µg/mL of PBA was not effective (Figure 2b). The cell viability was significantly affected due to PM stimulation and was successfully restored by the PBA treatment (Figure 2a). Fibroblasts expressed a similar trend (Figure 2c,d). Accordingly, with the results, concentrations except for 25 µg/mL were selected for subsequent experiments. NO levels or iNOS production was not evident during the study. A significant upregulation of the pro-inflammatory mediators including PGE_2_ (Figure 3d) and its modulator COX-2 was observed with the treatment against PM (Figure 4e,f). This was successfully downregulated via the treatment of PBA in keratinocytes. A similar trend was evident with the pro-inflammatory cytokines IL-1β and IL-6 (Figure 3a–c).

### 2.4. Potential of PBA to Abate PM-Induced Inflammatory Responses via NF-κB and MAPK Pathways in Keratinocytes

In the process of evaluating the activity of PBA as an anti-inflammatory agent, this research subsequently assessed whether or not the inhibition of inflammation responses are mediated via the NF-κB and MAPK pathways. The PM-induced phosphorylation of p38, ERK1/2, and JNK MAPKs in keratinocytes were considered via Western blotting. As illustrated (Figure 4a–d), PM encouraged the phosphorylation of NF-κB and MAPK mediators. PBA treatment (50, 100, and 200 µg/mL) gradually down-regulated the phosphorylation of p38, ERK1/2, and JNK. The results suggesting that PBA acts effectively upon p38 though all the MAPKs were significantly inhibited. Cytosolic p-50 and p-65 mediators were observed to be phosphorylated against PM induction and were successfully downregulated via the PBA treatment.

### 2.5. Effect of PBA on PM-Induced NF-κB and MAPK Proteins in Fibroblasts

**Figure 5:**
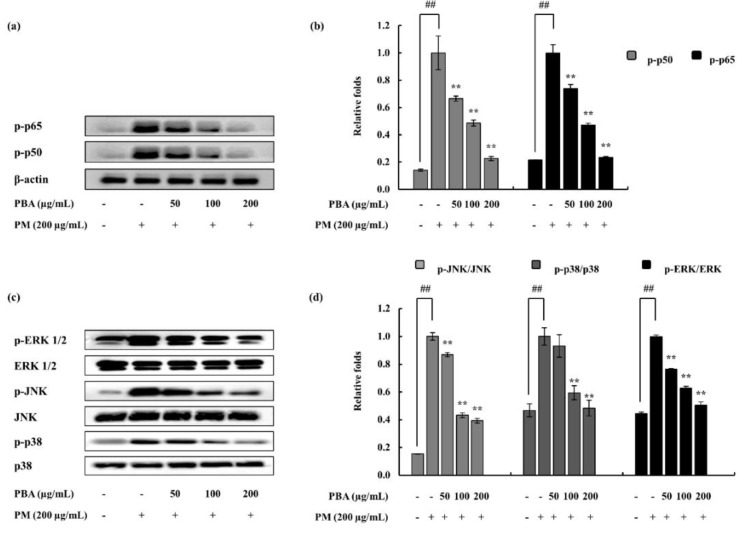
PM-induced HDF cells and co-treatment with PBA. (**a**) p50 and p65 in the cytosol, (**c**) p38, JNK, and ERK, data determined using Western blotting. Quantitative data were analyzed using ImageJ software (**b**,**d**). Results are expressed as the mean ± SD of three separate experiments. ** *p* < 0.01. (# denotes significance compared to control while * represents significance compared to PM treated group).

As illustrated in Figure 5a,b, cytosolic p50 and p65 phosphorylation were stimulated by PM. This was effectively and dose-dependently reduced by the PBA treatment. To determine fibroblasts, PM-induced inflammation is mediated via the MAPK pathway and to evaluate the effect of PBA, pathway proteins were assessed via Western blotting. Phosphorylation of JNK, p38, and ERK1/2 was upregulated via PM stimulation. However, PBA (50, 100, 200 µg/mL) reduced MAPKs in PM activated fibroblasts. Comparatively, PBA to act upon JNK was observed among the PBA’s significant potential.

### 2.6. Keratinocytes Stimulated with PM and Treated with PBA; Compositional Analysis

To analyze the metal ion composition of the samples which were treated with PBA following stimulation of keratinocytes with PM, ICP-OES was used. Several metal ions including Mg, Al, K, Ca, Fe, Mn, Cu, Sr, Ba, and Pb were observed to be significantly increased in the PM-treated group (Table 2). The record highest was Pb, while Cu remained the lowest. Our experiment suggested that, with the treatment of PBA, the metal concentrations downregulated dose-dependently. Substantial cutbacks were observed in Pb and Cu, followed by Sr, Ba, and Mg.

## 3. Discussion

Air pollution due to particulate matter has become a major concern in recent years. The East Asian region is reported to be highly affected. Contemporary publications suggest that particulate matter exposure is related to respiratory complications, allergic reactions, and inflammatory skin conditions. This is a complex mixture of different components. It includes various dust types such as tobacco smoke, pollen, and exhaust gas from traffic emissions [14].

The present study evaluated the physical parameters of the PM via the SEM and continued on inflammatory effects in keratinocytes and fibroblasts. Alginic acid was purified from *P. boryana* and its ability to inhibit PM-stimulated inflammation was evaluated. As indicated in Table 1, the purified alginate (PBA) consisted of a comparatively high amount of polysaccharide and traceable amounts of proteins and polyphenols. This supports the efficient purification of alginate. Initially, the *P. boryana* powder was depigmented using both hexane and 95% ethanol. This ensures the removal of lipids, pigments both non-polar and polar, as well as polyphenols reasonably. It is rather difficult to remove the effect of polyphenolic compounds due to their strong dipolar moments between polysaccharides. The usage of 10% formaldehyde in ethanol facilitates the formation of a phenolic polymer which in result lowers the solubility of phenolic substances and hence could be removed from the sample [15]. The alginic acid is presented in brown seaweed, prominently as calcium salt, but other forms such as magnesium, potassium, and sodium salts are also available in minute amounts. In this particular method, the alginates in the seaweed are converted into soluble alginate via alkaline treatment. Before this step to increase the extraction efficacy, the sample is acid washed with dilute mineral acid (HCl). The calcium ions are exchanged with the protons and at the same time, mineral acid removes the acid-soluble phenolic compounds. The following filtration step guarantees the removal of insoluble seaweed residues and the subsequent continuation of the sodium alginate solution. Recovering sodium alginate from this solution is not practical via evaporation due to its low concentration. Hence, the alginates can be precipitated as its calcium salt through the addition of CaCl_2_. The recovered alginates in the form of calcium alginates are then converted into alginic acid by the addition of dilute mineral acid. Finally, the alginic acid is further converted into sodium alginate using NaOH and the pH is uplifted to a neutral value [16]. The dialysis process removes excess ions. Alginic acid is mainly based on two monomeric units. Β-D-mannuronic acid and α-L-guluronic acid, which are respectively designated as M and G blocks. The polymer is formed via joining the monomers at C-1 and C-4 positions. The polymer chain consists of three kinds of molecules: M blocks based entirely on β-D-mannuronic acid, G blocks derived from α-L-guluronic acid, and MG blocks including interchanging units of the two acids. The proportion of the three types of blocks determine the physical properties of alginates [17,18,19]. Donati et al. (2003) reported that the monomer sequence is possible to differ not only among different species but also in variable tissues in the same species [20].

The chemical characterization of PBA is in good argument with the sodium alginate commercial sample. The study referred to several previously published data along with the analyzed data of this research. The broad peak at the 3425 cm^−1^ indicates the O-H stretching vibrations of the hydrogen bonds. The asymmetric O-C-O stretching vibrations of the carboxylate groups are represented via the 1680 cm^−1^ intense peak, while symmetric vibrations are indicated via the 1420 cm^−1^ intense band. Furthermore, a weak band at the 1035 cm^−1^ assigns C-O and C-C stretching vibrations in the pyranose ring. The anomeric carbons are represented in the 750–950 cm^−1^ region [13]. The interaction between metal ions and carboxylate groups in alginates in FTIR representation is further discussed in the report published by Papageorgiou et al. (2010) [21]

In between the external and internal environments, the skin is considered to be more than a physical barrier. A crucial role of the skin is to provide immune functions. It is suggested to function as a semiautonomous immunological organ. As the keratinocytes are the outermost layer of the skin, it is regularly used to assess the effect of the irritants in the dermatology. These cells participate in immune responses via the production of cytokines against the inflammatory events. This function widely contributes to the skin’s function as an immune organ. Keratinocytes can transfer stimuli into signals and successively to the other members of the skin immune system [9].

Heavy metal contamination is associated with biosorption, accumulation and toxicity; thus it has become a major concern that causes both environmental and health issues. The metal ion concentrations (Pb, Ca, Sr, Ba, and Mg) were significantly down-regulated dose-dependently via the treatment of PBA. Earlier reports by Schaumann et al. (2004) indicate that transition metal ions (Zn, Cu, and Cd) are responsible for the cause of inflammation via inducing oxidant generation. The report further illustrates that the increased concentrations of metal ions in the particulate matter contributes to the oxidative stress and hence promotes the activation of several transcription factors leading to discharge of pro-inflammatory mediators [22]. Heavy metals can be removed from a system using vivid methods according to Wang et al. (2011), one of them being the chemical precipitation of metal ions using potassium/sodiumthiocarbonate, sodium-dimethyl dithiocarbamate, and tri mercapto triazine. Some other methods are sulfide precipitation, adsorption, filtration using membranes, and ion exchange. Furthermore, the chelation of heavy metals using biopolymers now receiving much attention among the scientific community [23,24]. This follows the formation of complexes between the biopolymers and the metal ions. The affinity is influenced by several factors including the structure of the polymer and ionic charge, electronic configuration, and the coordination number of the metal ion [25]. Alginic acid as well as its derivatives are considered as capable polysaccharides in metal chelation [26]. As alginic acid contains carboxylic groups, metal carboxylate coordination can take place. Four distinct metal-carboxylate coordination types are described by Papageorgiou et al. (2010): ionic uncoordinated, unidentate coordination, bidentate chelating coordination, and bidentate bridging [21]. The structure of the alginic acid consists of M and G blocks and this influences the metal ion chelation. The “egg-box” model proposed by Grant et al. (1973) describes alginates as preferring to bind with divalent cations [27,28].

The inflammation process is regulated via complex signaling pathways. The process possibly initiated and developed involving several pro-inflammatory cytokines. The cytokines addressed in this research were downregulated upon the PBA treatment. Among them, IL-6 indicated significant downregulation compared to others. Furthermore, PGE_2_, another inflammatory mediator, was also declined. The COX-2 downregulation supported the PGE_2_ decrement as it is an enzyme involved in the generation of PGE_2_ via the arachidonic pathway. Downstream signals in the NF-κB and MAPK pathways were also investigated. Kim et al. (2014) address these signals as important regulators in inflammation studies focusing on cytokine-induced keratinocytes and skin. It reports the inhibition of JAK/STAT, NF-κB, and PI3K/Akt signaling to result in pro-inflammatory mediator, enzyme, cytokine, and chemokine inhibition [29]. The NF-κB dimers residing in the cytoplasm interact with the inhibitory proteins, IκBs. With the stimulation mainly due to pro-inflammatory cytokines, the IκB kinase (IKK) is activated and phosphorylation is initiated (p50 and p65). Hence, these are translocated to the nucleus to activate gene transcription. IKK is a complex formed from three distinct subunits with different functions: IKKα, IKKβ, and IKKγ. Among these, IKKβ is essential in NF-κB activation while IKKα is involved in the signal development process [30]. The present study indicated a significant downstream in the NF-κB-associated signals, confirming the effect of PBA against the stimulation of PM. Similarly, MAPK signals were also down-regulated, convincing the potential of PBA against the PM. MAPKs play an important role in inflammation via activating pro-inflammatory cytokines and chemokines [31,32]. These are a family of serine/threonine protein kinases that mediate biological processes in response to external stress signals. Out of the three main MAPKs (p38, JNK, and ERK), p38 MAPK signals are especially involved in the regulation of the synthesis of inflammatory regulators. These factors make MAPKs a potential target in anti-inflammatory therapeutics [33].

The study enhances the understanding of cellular mechanisms related to PM-induced inflammation that could result in effective drug development. PBA could be used as a potential candidate for the treatment of PM-stimulated skin damage. This simple model is applicable to evaluate the further effects of PM in the skin, such as intracellular and intercellular molecular cascades that lead to skin damage. As the skin is comprised of multiple layers of cellular components, the impact of the inflammation of the outermost layer can be assessed on other layers. Current research demonstrated the potential of *P. boryana* alginates to be implemented as a protective barrier against inflammation imposed with heavy metal and bacterial-derived endotoxins bound to the surface of the PM. Furthermore, in vivo studies could provide information concerning application methods, frequency of usage, and complex biological attributes accompanied.

## 4. Materials and Methods

### 4.1. Materials

The certified reference material, CRM No. 28 (Urban aerosols), was purchased from the Centre for Environmental Measurement and Analysis, National Institute for Environmental Studies, Ibaraki, Japan. The cell lines required for the experiments, HaCaT cells, and the human dermal fibroblast (HDF) were purchased from the Korean Cell line Bank (KCLB, Seoul, Korea). Dulbecco’s modified Eagle’s medium (DMEM), fetal bovine serum (FBS), and antibiotics (penicillin and streptomycin) for growth medium were purchased from the GIBCO Inc. (Grand Island, NY, USA). 3-(4,5-dimethylthiazol-2-yl)-2,5-diphenyltetrazolium bromide (MTT) was obtained from Sigma-Aldrich (St. Louis, MO, USA). Antibodies used in the Western blot analysis were from Santa Cruz Biotechnology (Santa Cruz, CA, USA). The cytokine kits used in the experiments were purchased from eBioscience (San Diego, CA, USA), R&D Systems (Minneapolis, MN, USA), BD Opteia (San Diego, CA, USA), and Invitrogen (Carlsbad, CA, USA). All the organic solvents used in the experiments were of analytical grade unless specified and were purchased from Sigma-Aldrich.

### 4.2. Alginic Acid Purification from P. boryana

*P. boryana* samples were collected from the coastal areas of Fulhadhoo Island, the Maldives. Samples were immediately washed with running water to remove salts and debris. The sample identification was assisted by Jeju Biodiversity Research Institute. Sample repositories were kept in the Laboratory of Marine Bioresource Technology at Jeju National University. Samples were then lyophilized and ground into a fine powder. Alginic acid extraction followed the method described by Fernando et al. (2018) with some minor modifications [28]. An initial depigmentation was carried out, first with hexane, and was followed by 95% ethanol. This was then soaked in 10% formaldehyde (in ethanol) for 10 h, filtered, and thoroughly washed with 95% ethanol to remove residual formaldehyde. The powder was air-dried and lyophilized. This was immersed in distilled water and the pH was adjusted to 4.0 using diluted HCl. The pH of the suspension was maintained at 4.0 during the whole step. The mixture was agitated at 30 °C for 24 h and was filtered and washed with distilled water. The sample was then soaked in 5% Na_2_CO_3_ (*w*/*v*) and agitated at 30 °C for 24 h. The resulting extract was filtered and the debris was clarified through centrifugation (10,000× *g*, 4 °C for 10 min). The recovered supernatant pH was adjusted to 6.0 by the addition of diluted HCl. This was treated with a saturated CaCl_2_ solution and alginic acid was precipitated as calcium alginate. The pellet was recovered via centrifugation and suspended in 10% HCl for 2 h (acid wash, 6×). Centrifugation was repeated to recover the pellet. Finally, the suspension was washed with distilled water and was neutralized using NaOH. The resulting alginate solution was extensively dialyzed and lyophilized to obtain *P. boryana* alginate powder (PBA).

### 4.3. Analysis of the Proximate Composition of P. boryana

The assessment included moisture, ash, protein, lipid, and polysaccharide contents. Moisture by drying at 100 °C, ashing in a furnace at 600 °C for 5 h, protein content via Kjeldahl digestion, soxhlet method for lipids and polysaccharides by phenol sulphuric method. The Association of Official Analytical Chemists standard methods (AOAC 1990), were implemented in analyzing the above of *P. boryana*.

### 4.4. Evaluation of Chemical Composition of PBA

Chemical composition evaluation of an extract is an essential step before proceeding to further biological and physical properties. This includes the assessment of polysaccharide, protein, and polyphenol content.

The polysaccharide content was evaluated via the phenol sulfuric method as described by DuBois et al. (1956) [34], with minor modifications. The method, in brief, including a calibration standard curve was plotted using d-glucose 0 to 0.1 mg/mL. Sample concentrations were maintained at 0.1 mg/mL. Phenol (80%), 25 µL, was treated into each tube. This was followed by the addition of 2.5 mL of conc. sulfuric acid and vortexed. The tubes were kept in the dark at an ambient temperature for 30 min. A volume of 200 µL from each tube was transferred to a 96 well plate and the absorbance was measured at 480 nm.

The polyphenol content was measured accordingly with the method described by Chandler and Dodds [35], with minor modifications. Gallic acid was used as the standard and a calibration curve was plotted (0 to 0.1 mg/mL). Samples were prepared to be 0.1 mg/mL. Each test tube was introduced with 1 mL of 95% ethanol, and was followed by 5 mL of distilled water and 0.5 mL of 50% (1N) Folin-Ciocalteau reagent. Tubes were vortexed and incubated for 1 h in a dark environment. Subsequently, a volume of 200 µL was transferred to a 96 well plate and the absorbance was measured at 700 nm.

Protein percentages were quantified using Pierce™ BCA Protein Assay Kit and bovine serum albumin used as the standard.

### 4.5. Functional Group Analysis of PBA Using FTIR

The powder method was used to analyze the functional groups via FTIR. Fourier-transform infrared spectroscopic (FTIR) analysis of the alginate was performed with a Thermo Scientific NicoletTM 6700 FTIR spectrometer (Thermo Fisher Scientific, Waltham, MA, USA). Potassium bromide (KBr) pellets were cast by combining a 5 mg sample with 5 g KBr powder. A fine powder was generated using a mortar and pestle. KBr pellets were cast by applying pressure to the mold (5000–10,000 psi). The pellets were then placed in the sample holder and scans (32) were collected within the range of 500–4000 cm^−1^ wavenumber having a resolution of 4 cm^−1^. A background scan was collected initially. The results were analyzed using the “Origin pro-2015” software package.

### 4.6. FTIR Spectra Interpretation Using Computational Calculations

Gaussian view molecular modeling software was used to develop the Cartesian coordinates for the Gaussian calculations. Initial energy calculations: geometry optimization of the molecule was performed using B3LYP quantum mechanical methods. The molecule was optimized finely and the harmonic vibrational frequencies were performed by ab initio time-dependent density functional theory (DFT) calculations at B3LYP level using the 6–31G (d,p) basis set as described by Cardenas-Jiron et al. (2011) [36] A scaling factor of 0.9645 was added to the calculated vibrational spectra.

### 4.7. Maintenance of Cell Lines

HaCaT cell line was maintained in the DMEM medium, which was supplemented with 10% FBS and 1% antibiotics. DMEM medium supplemented with F12 (25%), FBS (10%) and 1% antibiotics were used to maintain the human dermal fibroblast (HDF) cell line. The cells were maintained under controlled conditions at 5% CO_2_ level and 37 °C temperature. The cells were periodically subcultured and were used for experiments in their exponential growth phase.

### 4.8. Analysis of Cell Viability and Intracellular ROS

The cytotoxicity was evaluated using the MTT assay in PM-induced HaCaT and HDF cells. An MTT colorimetric assay was performed following the method described by Mosmann et al. (1983) with slight modifications [37]. The cells were seeded with a concentration of 1 × 10^5^ cells mL^−1^, in 24 well plates. After a 24 h incubation period, PBA with different concentrations was treated. The growth media DMEM was used to suspend the PM achieving a stock concentration. To obtain the treatment concentrations, a serial dilution was performed. PM was treated and given a 1 h incubation. Following a 24 h incubation period, MTT (2 mg/mL in PBS) was added and incubated for another 3 h. Then the medium was aspirated and the formazan crystals were dissolved in DMSO. The absorbance reading was taken at 540 nm. The optimum PM treatment concentration was selected via this method.

The intracellular ROS levels were evaluated using the DCF-DA assay. The cells were seeded, samples were treated, and following a 1 h incubation period, the cells were treated with a DCF-DA reagent. This was incubated for 10 min and the fluorescence intensity was determined at an excitation wavelength of 485 nm and an emission wavelength of 535 nm [38].

### 4.9. PGE_2_ and Pro-Inflammatory Cytokine Production Level Assessment

To obtain the cell culture media for the assessment of cytokine experiments, the cells were seeded in a similar manner described in the above experiments. HaCaT cells were seeded and incubated for 24 h. Samples were treated and after 1 h, PM was treated. After 23 h incubation, the media were retrieved for cytokine analysis. The culture media were collected separately and the expression levels of prostaglandin E2 (PGE_2_), tumor necrosis factor α (TNF-α), interleukin (IL)-1β, and IL-6 were measured. The process was assisted with commercially available cytokine assessing kits and the test was performed following the given instructions by manufacturers.

### 4.10. Western Blot Analysis

To identify several key molecular mediators, Western blot analysis was performed. The cells which were induced with PM were harvested within 30 min to analyze the upstream molecules in the MAPK and NF-κB pathway. A further 24 h incubation was continued to evaluate the COX-2 levels [28]. Ice-cold PBS was used to wash the harvested cells and was lysed using a nuclear and cytoplasmic protein extraction kit (NE-PER^®^, Thermo Scientific, Rockford, IL, USA). Each extract protein level was measured using a BCA protein assay kit and was standardized (Bio-Rad, Irvine, CA, USA). Electrophoresis was carried out using sodium sulfate-polyacrylamide gels (12%). Subsequently, transferred onto nitrocellulose membranes. The membranes were blocked with skim milk and were incubated overnight with relevant primary antibodies: β-actin, COX-2, p38, p-p38, ERK1/2, p-ERK1/2, JNK, p-JNK, p50, p-p50, p65, and p-p65 (Santa Cruz Biotechnology). After the incubation period, primary antibodies were removed and the HRP-conjugated secondary antibodies (anti-mouse IgG, Santa Cruz Biotechnology) were added. Then, signals were developed using the chemiluminescent substrate (Cyanagen Srl, Bologna, Italy). Membranes were photographed (FUSION SOLO Vilber Lourmat system) and the ImageJ program was assisted in the quantification of the band intensities [39].

### 4.11. Spectroscopic Analysis (ICP-OES)

Similarly, HaCaT cells were seeded and samples were treated. PM was treated following an incubation period. After the procedure, the cells were harvested and collected into pre-weighed tubes. Cells were dried, and the final weight was taken. This was digested in concentrated HNO_3_ using thermal energy (10% H_2_O_2_ was added). The acid digests were diluted in 3% HNO3 acid. The metal ions were analyzed by inductively coupled plasma optical emission spectrometry (ICP-OES) system (PerkinElmer OPTIMA 7300DV, Inc., Waltham, MA, USA). The calibration curves were plotted using a multi-element standard (PerkinElmer N9300233) including 10 ppm (10 µg/mL) of each element (Mg, Al, K, Ca, Fe, Mn, Cu, Sr, Ba, Pb). The elements were detected by non-overlapping wavelengths (≥2) [11]. Ultrapure deionized water was used in each step of the experiment. The final concentrations of the samples were calculated concerning the calibration plots.

### 4.12. Statistical Analysis

Based on triplicated experiments, data are expressed as means ± SD. One-way ANOVA and Turkey’s test was used to determine the significant differences among data values. A *p* < 0.05 was considered statistically significant. * *p* < 0.05 and ** *p* < 0.01. (# denotes significance compared to control while * represents significance compared to PM-treated group).

## 5. Conclusions

The anti-inflammatory effects of alginic acid purified from *P. boryana* are evident in this research. Particulate matter induced inflammation in keratinocytes, as well as fibroblasts, were inhibited via the activity of PBA. Even though it requires further experimental confirmation, the researchers believe the inflammation was highly encouraged due to the effects of heavy metal content in the PM. Hence, PBA successfully chelated the metal ions, reducing their concentrations in the cell digests. Thus, PBA, a natural bioactive, is applicable as a source of skin cosmetics to abate PM-induced inflammation.

## Figures and Tables

**Table 1:** Chemical composition of purified alginic acid from *P. boryana.*

Composition	Content (%)
Polysaccharide	79.84 ± 1.32
Ash	3.42 ± 0.56
Protein	1.22 ± 0.18
Polyphenol	2.17 ± 0.69
Yield	16.85 ± 0.32

All results expressed as means ± SE, based on triplicated trials.

**Table 2:** Metal composition analysis.

	Control	PM	PM + PBA (50)	PM + PBA (100)	PM + PBA (200)
Mg	54.67 ± 1.47	128.53 ± 2.51	137.44 ± 4.97	101.46 ± 6.14	84.95 ± 2.11
Al	0.91	125.08 ± 9.1	119.33 ± 8.21	79.31 ± 5.12	56.92 ± 3.52
K	452.98 ± 14.56	437.37 ± 18.47	522.65 ± 20.22	504.28 ± 31.58	466.12 ± 19.68
Ca	224.44 ± 12.56	344.37 ± 10.45	385.24 ± 9.56	321.07 ± 5.36	256.44 ± 7.48
Fe	ND	174.50 ± 4.57	163.62 ± 6.89	106.52 ± 7.58	77.27 ± 5.23
Mn	ND	80.2 ± 2.47	68.4 ± 3.93	45.2 ± 2.42	32.1 ± 2.9
Cu	ND	20.1 ± 1.44	10.84 ± 1.89	6.53 ± 2.01	ND
Sr	0.71	42.18 ± 1.32	31.94 ± 2.58	21.37 ± 1.22	11.86 ± 1.09
Ba	ND	92.92 ± 6.33	72.56 ± 4.39	51.99 ± 3.15	20.41 ± 1.02
Pb	ND	241.74 ± 12.46	168.41 ± 14.15	102.47 ± 6.27	58.96 ± 3.68

All results expressed as means ± SE, based on triplicated trials. ND—Not detected. Mg; Magnesium, Al; Aluminium, K; Potassium, Ca; Calcium, Fe; Iron, Mn; Manganese, Cu; Copper, Sr; Strontium, Ba; Barium, Pb; Lead.
